# Similar Transition Processes in Synovial Fibroblasts from Rheumatoid Arthritis and Osteoarthritis: A Single-Cell Study

**DOI:** 10.1155/2019/4080735

**Published:** 2019-07-24

**Authors:** Shaozhe Cai, Bingxia Ming, Cong Ye, Shengyan Lin, Peng Hu, Jungen Tang, Fang Zheng, Lingli Dong

**Affiliations:** ^1^Department of Rheumatology and Immunology, Tongji Hospital, Huazhong University of Science and Technology, No. 1095 Jiefang Avenue, Wuhan 430030, China; ^2^Department of Immunology, School of Basic Medicine, Tongji Medical College, Huazhong University of Science and Technology, Wuhan 430030, China

## Abstract

Rheumatoid arthritis (RA) and osteoarthritis (OA) are common rheumatic disorders that primarily involve joints. The inflammation of the synovium can be observed in both of the two diseases. Synovial fibroblasts (SFs) play an important role in the inflammatory process of the synovium. The functional states of synovial fibroblasts are heterogeneous, and the detailed transition process of their functional states is still unclear. By using transcriptomic data of SFs at a single-cell level, we found a similar transition process for SFs in RA and OA. We also identified the potential regulatory effects of the WNT signaling pathway, the TGF-*β* signaling pathway, the Fc*ε*RI signaling pathway, and the ERBB signaling pathway on modifying the SFs' functional state. These findings indicate potentially overlapped pathogenic mechanisms in these two diseases, which may help uncover new therapeutic targets to ameliorate disease progression.

## 1. Introduction

**Figure 1:**
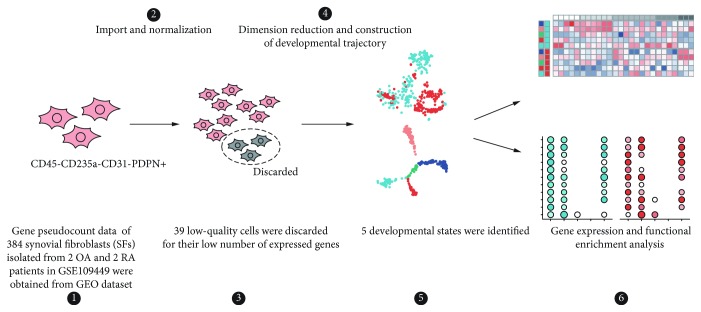
Overall design of this study. Firstly, scRNA-seq data of 384 CD45-CD235a-CD31-PDPN+ synovial fibroblasts were downloaded from GEO dataset (GSE109449, Ref. [5]). After data import and normalization, 39 low-quality data were discarded. Then, dimension reduction was made and developmental trajectory was constructed, and 5 developmental states were identified. DEGs and functional enrichment analyses were made to illustrate the similarity and heterogeneity of SFs between RA and OA, and among different states.

Rheumatoid arthritis (RA) and osteoarthritis (OA) are common rheumatic disorders that primarily involve joints. RA is an autoimmune disease, and an immune-mediated etiology associated with stromal tissue dysregulation can together propagate chronic inflammation and articular destruction [1]. OA has long been viewed as a degenerative disease of the cartilage, but accumulating evidence indicates that inflammation has a critical role in its pathogenesis [2]. The synovium consists of the synovial lining and the subjacent vascular and areolar tissue up to the capsule. Synovial fibroblasts (SFs) are dominant cells in normal synovium [3]. In synovitis, SFs play an important role in the local immunoinflammatory responses [2, 4]. Recently, several studies revealed the existence of different subsets of synovial fibroblasts [5, 6]. Insights into the transition process of synovial fibroblasts can help us better understand the pathophysiological role of SFs in both RA and OA. However, no study has explored the transitional process of the synovial fibroblasts of these 2 diseases in vivo in humans. Here, by using the single-cell RNA-sequencing data of RA and OA synovial fibroblasts (Figure 1), we aim to explore the potential transition process of SFs in vivo and elucidate their corresponding functional states.

## 2. Materials and Methods

### 2.1. Single-Cell mRNA Sequencing Data

Gene pseudocount data (reported by Kallisto 0.42.4) of 384 CD45-CD235a-CD31-podoplanin (PDPN) + synovial fibroblasts (SFs) in GSE109449 were downloaded from the Gene Expression Omnibus Dataset (GEO Dataset: http://www.ncbi.nlm.nih.gov/geo/). From a total of 384 SFs, 192 SFs were obtained from OA patients, while the other 192 SFs were obtained from RA patients. Expression levels of mRNA in this dataset were assayed with the Smart-Seq2 protocol [5].

### 2.2. Data Reprocessing and Quality Control

Pseudocounts of genes were imported and normalized with the R package tximport [7]. The numbers of expressed genes (with at least 1 read count) were calculated for all 384 SFs. 39 low-quality cells were discarded because the numbers of their expressed genes were smaller than the medians of all cells minus 3 × median absolute deviation (MAD); thus, 345 cells remained (Supplementary Figure 1).

### 2.3. Data Analysis

Differently expressed genes (DEGs) were analyzed with the R package limma [8]. Dimension reduction via *t*-distributed stochastic neighbor embedding (*t*-SNE), unsupervised clustering, and developmental trajectory construction was realized with the R package Monocle [9]. Gene set enrichment analysis (GSEA) was analyzed with the Java-based GSEA Desktop program (version 2.2.4) [10, 11]. Normalized counts of genes were used for dimension reduction, developmental trajectory construction, and GSEA analysis. Log2-transformed normalized counts of genes were input to get DEGs. For DEG detection, genes with a *p* value <0.01 and a ∣log2FoldChange∣ (∣logFC∣) value > 1.5 were regarded as DEGs. For GSEA analysis, terms with NOM *p* val <0.05, FDR *q* val <0.25, and ∣NES∣ > 1 were regarded as significantly enriched.

Heatmaps in this study were plotted with the R package pheatmap (version 1.0.10). Other plots were drawn with the R package ggplot2 (version 3.1.0) or ggpubr (version 0.2).

## 3. Results

### 3.1. RA and OA SFs Show Similar Developmental Trajectory

**Figure 2:**
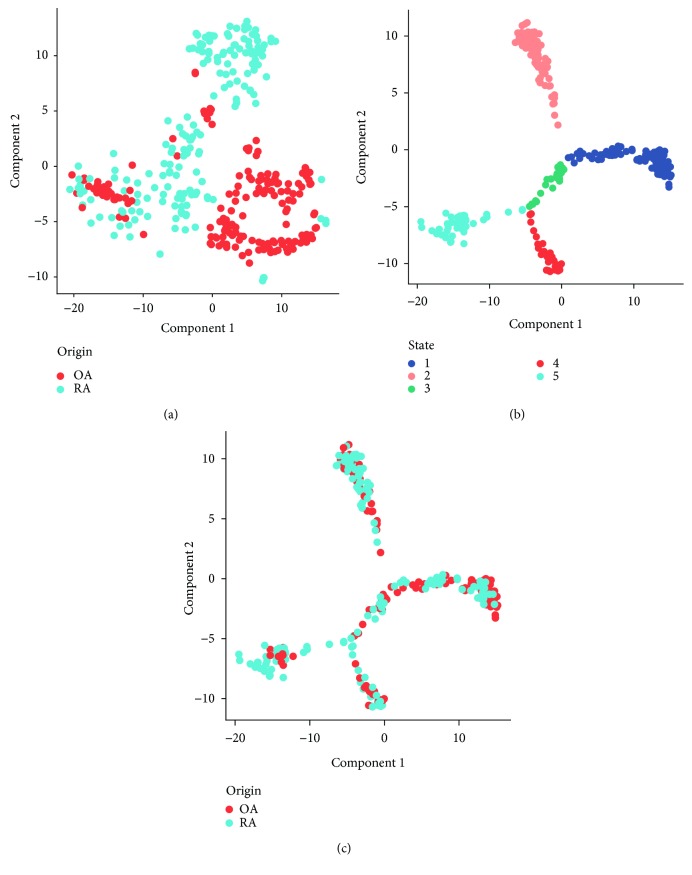
Clustering and developmental trajectory of RA and OA synovial fibroblasts. (a) *t*-SNE visualization of SFs from RA and OA patients. OA SFs: red; RA SFs: blue. (b) Developmental trajectory constructed by the R package Monocle. Different colors represent different states recognized by Monocle. (c) Distribution of SFs' origin in the developmental trajectory. SFs from OA (red) and SFs from RA (blue) show similar distribution patterns.

In order to address the similarity and heterogeneity among synovial fibroblasts with RA and OA origins, we firstly used the *t*-SNE method to visualize their distribution (Figure 2(a)). Unsupervised clustering showed potentially different cell clusters (Supplementary Figure 2). The majority of the cells were distributed in different areas with origin predisposition, while colocalization of several RA and OA SFs can also be observed. These two phenomena indicated that, besides the existence of heterogeneity in SFs, several SFs in RA and OA may share similarities. The heterogeneity of SFs may represent their underlying different functional status. To understand the transition of SFs' functional status, we applied the unsupervised inference method Monocle [9] to construct the potential developmental trajectories of SFs. Five states can be identified with Monocle (Figure 2(b)), and SFs from RA and OA showed a similar pattern of developmental trajectory (Figure 2(c)).

### 3.2. RA and OA SFs Show Similar Development Orientation Regardless of Anatomical Localization

**Figure 3:**
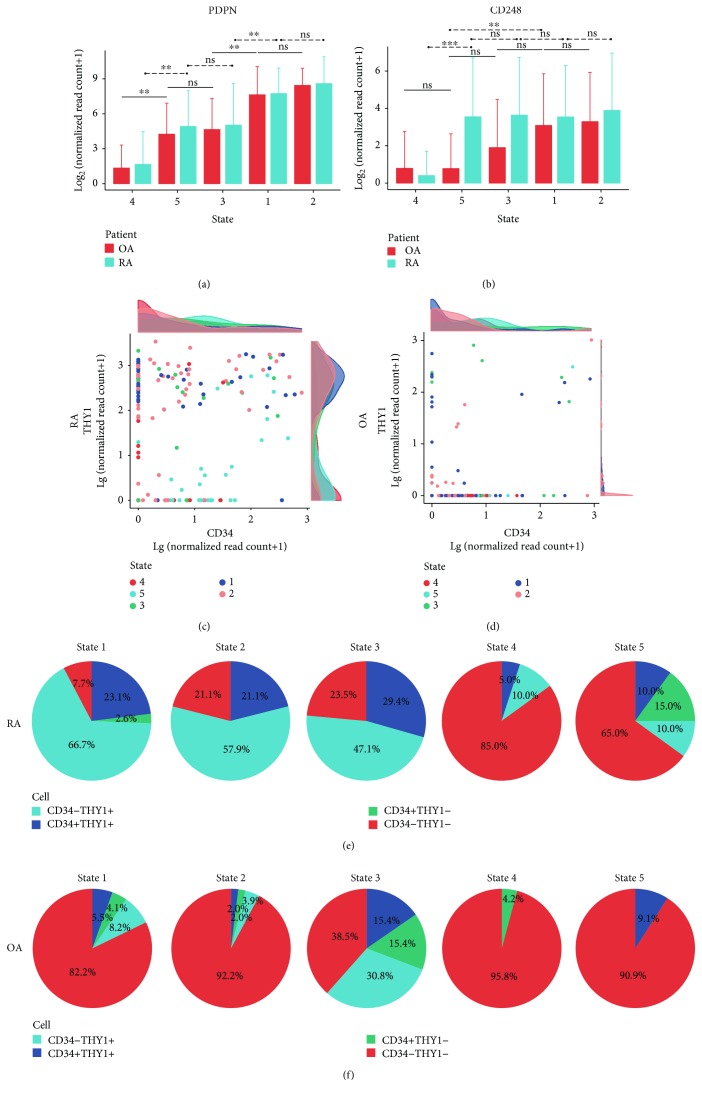
Prediction of transition orientation and localization of SFs. Average expression level (log2 transformed) of PDPN (a) and CD248 (b) in SFs of states 1 to 5. OA SFs: red; RA SFs: blue. Expression level of CD34 and THY1 in RA (c) and OA (d) SFs. Plots represent SFs. Density plots show the distribution of expression level (log10 transformed) of CD34 (upper) and THY1 (right) in the corresponding plot. Different colors represent different states. Composition of cell subsets divided by CD34 and THY1 across states 1 to 5 in both RA (e) and OA (f) SFs. Blue: CD34-THY1+; dark blue: CD34+THY1+; green: CD34+THY1-; red: CD34-THY1-. CD34+/THY1+: lg(normalized read counts of CD34/THY1 + 1) ≥ 1.5. Bars show means, and error bars show standard deviations. ^∗^ *p* < 0.01, ^∗∗^ *p* < 0.001, and ^∗∗∗^ *p* < 0.0001; ns = not significant.

PDPN and CD248 are surface markers of synovial fibroblasts. In vitro, the expression level of PDPN in synovial fibroblasts increases after stimulation with TNF*α* and IL-1*β*, while that of CD248 increases after stimulation with TGF-*β* [6]. In order to investigate the orientation of the developmental trajectory, we compared the gene expression levels of PDPN and CD248 in each state. For PDPN, the lowest expression level was observed in state 4, while the highest was observed in state 2 or state 1, in both OA and RA SFs (Figure 3(a)). The expression level of PDPN in state 3 or state 5 was also comparable. For CD248, the lowest expression level was also observed in state 4 (Figure 3(b)). In RA SFs, CD248 levels were similar across states 1, 2, 3, and 5, while in OA SFs, CD248 levels were comparable between state 3, state 4, and state 5. Thus, SFs in state 4 might be unstimulated, while SFs in the other states represented cells that had been influenced by inflammatory microenvironments. Based on the results from Ref. [6] which showed that the expression level of PDPN and CD248 on SFs are time-dependent, we assumed that the potential transitional process of SFs may have 2 branches based on the developmental trajectory pattern presented in Figure 2(b): from state 4 to state 5 or from state 4 to state 3 to state 1 or state 2. SFs in state 1 and state 2 may possess a higher invasive and destructive ability compared with SFs in states 3 or 5.

According to the study from Brenner et al., CD34-THY1+ fibroblasts in RA and OA were observed in sublining areas, and CD34-THY1- fibroblasts were mostly observed in lining areas, while CD34+ fibroblasts were observed in both superficial lining and deeper sublining areas of the synovium [5]. In order to understand the potential anatomical predisposition of SFs in different states with different origins, we further analyzed their expression level of CD34 and THY1 (Figures 3(b)–3(f)). SFs from RA and OA showed different patterns of CD34 and THY1 expression: more cells in RA expressed higher levels of CD34 or THY1, while in SFs from OA, the majority of the cells were CD34-THY1- (Figures 3(c) and 3(d)). SFs that expressed CD34 and/or THY1 could be observed across states 1 to 5, while most CD34-THY1+ cells were observed in states 1 to 3, particularly in RA SFs (Figures 3(e) and 3(f)). These results indicated that SFs in a different state could be observed in both the lining and sublining areas of the synovium, while the localization pattern of SFs in each state might vary between RA and OA.

### 3.3. Similarities and Heterogeneities among Different States Revealed by GSEA Analysis

**Figure 4:**
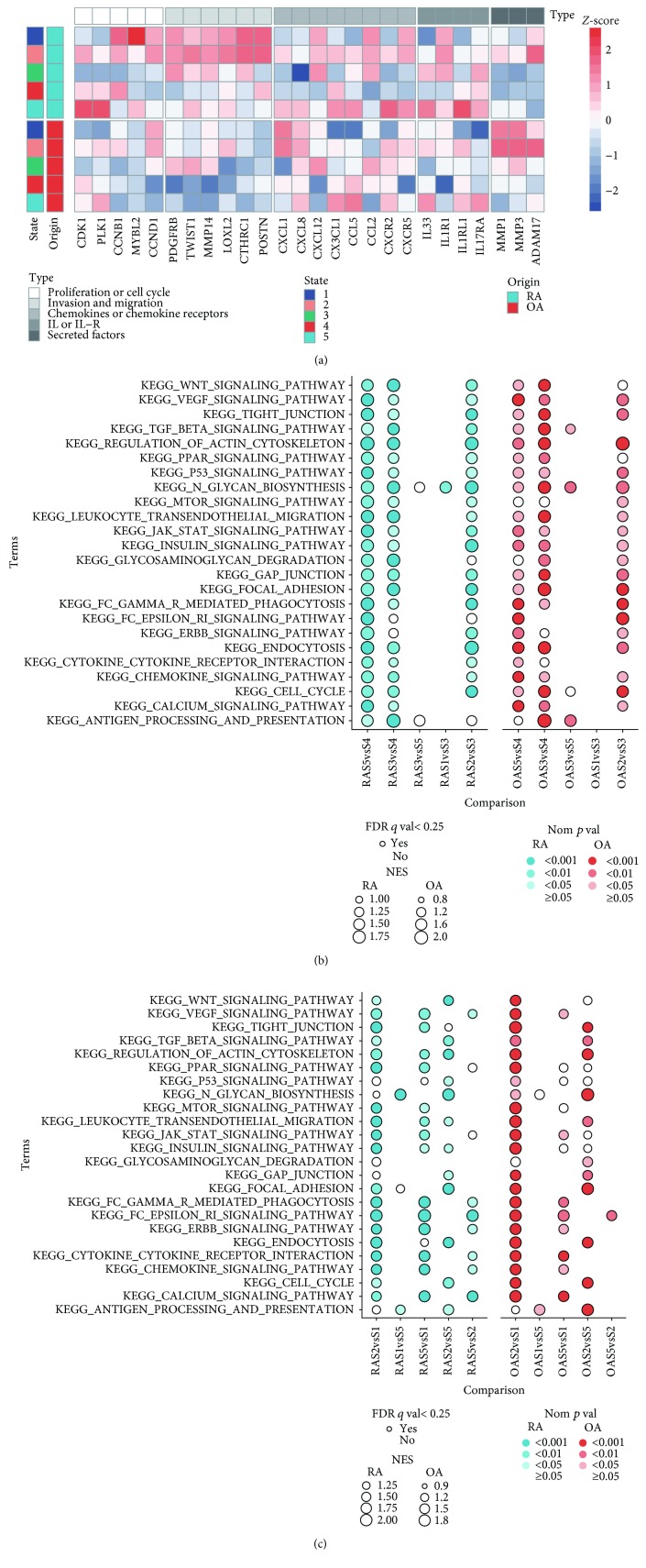
Similarity and heterogeneity among different states in RA and OA SFs. (a) Gene expression pattern of key effector molecules to predict the function of fibroblast subsets. Expression level of each gene is presented with *Z*-score. (b) Enriched KEGG terms via GSEA analysis along the assumed transition process. (c) Enriched KEGG terms via GSEA analysis among states 1, 2, and 5. In (b) and (c), *Origin*S*a*vsS*b* means the enrichment state of the corresponding term in state a when compared with state b in Origin SFs. For example, RAS2vsS1 means the terms enriched in state 2 when compared with state 1 in RA SFs.

Compared with synovial fibroblasts in state 4, SFs in states 1, 2, 3, and 5 showed stimulated characters. We first selected several key genes related to several biological processes based on reported researches to view the functional patterns of all these 5 states [5, 6, 12–15]. Genes that favor invasion and migration were expressed at the highest levels in state 1 and state 2, while genes of some proinflammatory interleukins and interleukin receptors had the predisposition to express more in state 5 (Figure 4(a)). For genes related to chemotaxis, higher levels of CXCL12 were observed in states 1, 2, and 3, and similar expression levels of CXCR2 were observed between states 2 and 5 (Figure 4(a)). We also focused on the gene expression pattern of major histocompatibility complex (MHC) molecules, toll-like receptors (TLRs), and adhesion molecules: TLRs and MHC class II molecules were highly expressed in states 2 and 5, while the expression level of MHC class I molecules were higher in states 1 to 3; ICAM1 and VCAM1 were higher in SFs of states 1 to 3, while ICAM2 was higher in state 5 (Supplementary Figure 3). These results supported our previous assumption that SFs in state 1 and state 2 may possess a higher invasive and destructive ability compared to SFs in states 3 or 5, and it also indicated the potential of SFs in different states to interact with different types of cells.

Next, we applied gene set enrichment analysis to help us elucidate the different functional states along the previously assumed transition process. Considering the impossibility to list all known pathways or biological processes, we selected 24 Kyoto Encyclopedia of Genes and Genomes (KEGG) terms related to several important pathophysiological pathways to present the functional similarity and heterogeneity of SFs. In order to simplify the illustration, we divided the assumed transition process into 2 parts: part 1 consisted of the transition from state 4 to states 3 or 5, while part 2 consisted of the transition from state 3 to state 1 or 2. Part 1: compared with state 4, nearly all these 24 terms were enriched in both state 3 and state 5, except that the enrichment of the Fc*ε*RI signaling pathway and the ERBB signaling pathway existed only in state 5 (Figure 4(b)). Although terms related to invasive capacity, like the WNT signaling pathway and the TGF-*β* signaling pathway, were enriched in both states 3 and 5, higher NES and lower nom *p* value were observed in state 3 [16]. SFs in state 5 enriched terms about enhancing inflammatory process (the terms calcium signaling pathway, Fc*ε*RI signaling pathway, and ERBB signaling pathway, and the term VEGF signaling pathway) (Figure 4(b), Supplementary Figure 4A) [17–19]. Part 2: compared with state 3, nearly all terms were enriched in state 2, while fast no terms were enriched in state 1 (Figure 4(b)). Interestingly, the WNT signaling pathway and the TGF-*β* signaling pathway were only enriched in the transition from states 3 to 2 in RA.

Comparisons among states 1, 2, and 5 were also made. Compared with state 1, most terms were enriched in state 2 and state 5 (Figure 4(c)). The difference between state 2 and state 5 is that SFs in state 2 enriched more terms related to the ability of invasion and proliferation (the terms WNT signaling pathway and the TGF-*β* signaling pathway and the term cell cycle). For RA SFs, the Fc*ε*RI signaling pathway, the ERBB signaling pathway, the calcium signaling pathway, the VEGF signaling pathway, the chemokine signaling pathway, and the cytokine-cytokine receptor interaction were enriched in state 5, while for OA SFs, only the Fc*ε*RI signaling pathway was enriched in state 5 (Figure 4(c)).

These results indicated that SFs in state 2 and state 5 were more pathogenic compared with SFs in states 1 and 3. SFs in state 5 possessed stronger proinflammatory ability, while SFs in state 2 were more invasive. The enriching predisposition of the Fc*ε*RI signaling pathway, the ERBB signaling pathway, the calcium signaling pathway, and the VEGF signaling pathway in state 5 and the WNT signaling pathway and the TGF-*β* signaling pathway in state 3 indicated that these signaling pathways may contribute to the branching of the transition process, which would lead to different functional phenotypes of SFs.

Although SFs from different origins showed a similar enrichment pattern in the comparisons above, the functional state of SFs from OA and RA in each state were not identical. When compared with OA SFs, genes related to antigen processing and presentation, cytokine-cytokine receptor interaction, and focal adhesion were enriched in RA SFs in states 1, 2, and 3, and the WNT signaling pathway and the TGF-*β* signaling pathway were enriched in RA in states 1 and 2 (Supplementary Figure 4B). The gene expression level of many proinflammatory proteins were also lower in OA SFs (Figure 4(a)). These indicated that stimulated SFs in RA might have received stronger stimulation and also exhibit stronger proinflammatory and invasive ability. Genes related to cell survival (e.g., mTOR signaling pathway) also had a predisposition to be enriched in several states in RA. No terms were enriched in state 3 and state 4. These results reflected the low-grade inflammation in OA.

## 4. Discussion

Compared with gene expression studies that use bulk RNA samples and provide only a virtual average of the mix of cells, single-cell studies enable the molecular distinction of all cell types within a complex population composition which can contribute to improve the understanding of how histologically identical, adjacent cells make different differentiation decisions during development [20]. Here, we focused on synovial fibroblasts, which are important components of the synovium and may exert important effects in the pathogenesis of RA and OA.

**Figure 5:**
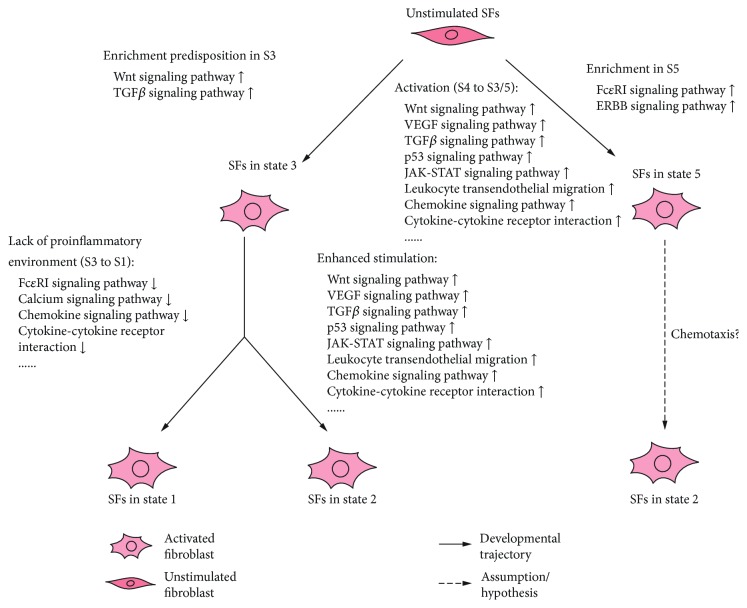
Enrichment of KEGG terms along the developmental trajectory. GSEA analysis revealed that several biological processes may take part in the transition of SFs from state 4 to states 1, 2, 3, or 5, and different combinations of these biological process in different active states may lead to distinct transition processes. S, state; ↑ enhanced; ↓ attenuated.

Understanding the transition process of SFs may help identify the pathological subsets of SFs and reveal the potential targets to help the treatment of RA and OA. Our study revealed a similar transition process in both RA and OA SFs: from an unstimulated state (state 4) to activated states with different functional patterns (states 1, 2, 3, and 5). The elevated expression level of PDPN can be observed after stimulation with TNF*α* or IL-1*β* in a time-dependent manner [6]. The lower expression level of PDPN in state 3 and state 5 indicated that SFs in these 2 states seem to be the “progenitor” of SFs in states 1 and 2. GSEA analysis revealed that most terms related to inflammation responses were enriched in state 3 when compared with state 1, while the other terms were enriched in state 2 when compared with state 3. These pointed out that the lack of proinflammatory environments may lead to the transition of SFs from state 3 to state 1, while enhanced stimulation may lead to the development to state 2. Interestingly, SFs in state 2 and state 5 seem to be highly activated; while SFs in state 2 showed a higher invasive capability, SFs in state 5 might exert stronger proinflammatory effects. Compared with SFs in state 2, the lower expression level of ICAM1 and VCAM1, the higher expression level of ICAM2 (similar in OA), and the enrichment of the Fc*ε*RI signaling pathway and the ERBB signaling pathway could be observed in SFs in state 5. The high expression pattern of TLRs could be observed in SFs of both state 5 and state 2. ICAM1 and VCAM1 are essential for the interaction between SFs and T/B lymphocytes, while the stimulation of SFs inducted by CD28- T cells is primarily mediated by ICAM2, not ICAM1 [15, 21–23]. The ERBB signaling pathway is essential for the signaling of several TLRs in some conditions [24, 25]. The Fc*ε*RI in DCs and monocytes may contribute to allergic diseases via enhancing T cell immunity and inflammation [19]. All these indicated that SFs in state 5 might be a subset of SFs with a stronger capacity to interact with resting T cells, and TLRs, the Fc*ε*RI signaling pathway, and the ERBB signaling pathway may participate in the stimulation of SFs in this state, while SFs in state 2 might have more capacity to interact with activated T and B cells. A similar expression pattern of CXCR2 between SFs in states 2 and 5 indicated that SFs in state 5 might have the potential to be recruited to the surrounding SFs in state 2 and be further influenced by the microenvironment promoting transition to state 2 (Figure 5).

Heterogeneity between RA SFs and OA SFs was also observed. Besides lower grade inflammation in OA, another obvious difference between SFs from OA and RA is the heterogeneity of composition and anatomical localization of SFs among different states. In RA, the ratio of the CD34-THY1+ SF subsets were significantly elevated in states 1 to 3, while most SFs in OA were lining cells (CD34-THY1-), which may verify the results that CD34-THY1+ SFs might be important subsets with pathological behavior in rheumatoid arthritis [5]. However, in our study, CD34-THY1+ cells could be observed in all transition states in RA. These pointed out the underlying heterogeneity in CD34-THY1+ SFs, while also raising the following question: Where did the CD34-THY1+ cells in the sublining area of the RA synovium come from? Several studies have revealed that CD34-THY1+ cells in the RA synovium may possess characteristics of mesenchymal stem cells (MSCs), which also shared similarity with MSCs with bone marrow origin (BM-MSCs) [26]. It is possible that the expanding synoviocyte population resulted from the migration of mesenchymal stem cells from the circulation or from the expansion of a stem cell pool in the synovium [27–29]. The enrichment of genes related to the WNT signaling pathway and the TGF-*β* signaling pathway along the transition branch from state 4 to state 2 or 1 in RA indicated the potentially high TGF-*β* level in the surrounding environments of these SFs (Figure 5). TGF-*β* can mediate the migration of MSCs from the peripheral blood or surrounding tissue to be integrated into the injured tissues, and accompanied with the WNT signaling pathway, can promote the proliferation of MSCs [30]. Thus, one reason for the accumulation of CD34-THY1+ cells in the sublining area of the synovium in RA might be the expansion of recruited MSCs from circulation, partly mediated by the TGF-*β* and WNT signaling pathways. CD34+ cells from bone marrow can be the progenitor of CD34-THY1+ MSC via culture in vitro, and the transition of the expression pattern from CD34+ to CD34-THY1+ in fibroblasts could also be observed in dermal fibroblasts in several diseases [31, 32]. Thus, the transition from CD34+ cells might be another origin of the CD34-THY1+ cells. However, the CD34- MSCs in Ref. [32] were derived mainly from CD34- populations. Thus, another question is whether there is a possibility that CD34-THY1+ cells were transited from CD34-THY1- cells or CD34+ cells. WGCNA analysis revealed that the coexpression pattern of genes related to MHC class I molecules mediated the antigen presentation process and several infectious processes to the expression of CD34 and THY1 (data not shown). However, we cannot figure out the causal relationship between them. Cells from different origins may respond to a similar stimulus in different ways. Thus, the heterogeneity of the proinflammatory microenvironment and the potential heterogeneity of the SFs' origins can both contribute to the heterogeneity between SFs from RA and OA patients. These can also point out the inappropriateness of the concept that SFs from OA can be regarded as a control of SFs from RA, which has existed in several related studies.

There are still some limitations in our study. Although the scRNA-seq data of 345 SFs were used in this analysis, they came from only 4 patients, which may lead to results biased by the individual characteristics of the limited number of patients. Also, the detailed effects of the pathways previously described need to be verified.

In conclusion, our study revealed the similar transition processes of SFs in both RA and OA, described the corresponding changes of the functional states in the transition process, and indicated the potential regulatory effects of the WNT signaling pathway, the TGF-*β* signaling pathway, the Fc*ε*RI signaling pathway, and the ERBB signaling pathway on the transition of synovial fibroblasts in both RA and OA, which may provide potential therapeutic targets to both conditions.

## Data Availability

Previously reported RNA-seq data of synovial fibroblasts at the single-cell level were used to support this study and are available at the webpage (https://www.ncbi.nlm.nih.gov/geo/query/acc.cgi?acc=GSE109449). This dataset is cited at relevant places within the text as references [5].

## Supplementary Materials

Supplementary Materials Supplementary Figure 1: assessing the quality of single-cell RNA-seq data. The numbers of the expressed gene (with at least 1 read count) were calculated for all 384 SFs. 39 low-quality cells were discarded because their number of expressed genes were smaller than the threshold (dash line, medians of all cells minus 3 × median absolute deviation), and 345 cells remained. The color of the plot represents its corresponding origin documented by GSE109449. Supplementary Figure 2: unsupervised clustering showing different potential cell clusters. This is based on the study from Rodriguez and Laio (Rodriguez, A. and A. Laio, *Machine learning. Clustering by fast search and find of density peaks.* Science, 2014. 344(6191): p. 1492-6). On the left panel, for each data point, *ρ* represents local density and *δ* represents its distance from points of higher density. On the right panel, the *γ* value (*γ* = *ρδ*) of each point on the left panel were plotted in decreasing order. Supplementary Figure 3: gene expression pattern of major histocompatibility complex (MHC) molecules, toll-like receptors (TLRs), and adhesion molecules across states 1 to 5 in RA and OA SFs. The higher expression level of TLRs and MHC class II molecules were observed in states 2 and 5, while that of the MHC class I molecules were observed in states 1 to 3; ICAM1 and VCAM1 were higher in SFs of states 1 to 3, while ICAM2 was higher in state 5 (for OA, in both state 2 and state 5). Supplementary Figure 4: GSEA analysis among states 1, 3, and 5 (A) and between RA and OA SFs across states 1 to 5 (B). (A) Similar enrichment patterns in state 5 were observed in both RA and OA SFs. The VEGF signaling pathway, the Fc*γ* and Fc*ε*RI signaling pathway, the ERBB signaling pathway, the calcium signaling pathway, the chemokine signaling pathway, and the cytokine-cytokine receptor interaction were enriched in state 5 in both RA and OA. In OA, terms related to proinflammatory and invasive capacity were enriched in SFs in state 3, while in RA, only genes related to the Fc*ε*RI signaling pathway, the calcium signaling pathway, the chemokine signaling pathway, and the cytokine-cytokine receptor interaction were enriched in state 3. The JAK-STAT signaling pathway was enriched in state 5 in RA and in state 3 in OA when compared with state 3 and state 2, respectively. (B) Except for the fact that no terms were enriched in RA SFs in state 3 and state 4, the enrichment pattern in RA SFs reflected a high-grade inflammation status in the RA synovium.
