# Supplementary material for: Similar Transition Processes in Synovial Fibroblasts from Rheumatoid Arthritis and Osteoarthritis: A Single-Cell Study
Source: J Immunol Res. 2019 Jul 24;2019:4080735. doi: 10.1155/2019/4080735 (PMC6681591; doi:10.1155/2019/4080735)
Supplement: Supplementary Materials — Supplementary Figure 1: assessing the quality of single-cell RNA-seq data. The numbers of the expressed gene (with at least 1 read count) were calculated for all 384 SFs. 39 low-quality cells were discarded because their number of expressed genes were smaller than the threshold (dash line, medians of all cells minus 3 × median absolute deviation), and 345 cells remained. The color of the plot represents its corresponding origin documented by GSE109449. Supplementary Figure 2: unsupervised clustering showing different potential cell clusters. This is based on the study from Rodriguez and Laio (Rodriguez, A. and A. Laio, Machine learning. Clustering by fast search and find of density peaks. Science, 2014. 344(6191): p. 1492-6). On the left panel, for each data point, ρ represents local density and δ represents its distance from points of higher density. On the right panel, the γ value (γ = ρδ) of each point on the left panel were plotted in decreasing order. Supplementary Figure 3: gene expression pattern of major histocompatibility complex (MHC) molecules, toll-like receptors (TLRs), and adhesion molecules across states 1 to 5 in RA and OA SFs. The higher expression level of TLRs and MHC class II molecules were observed in states 2 and 5, while that of the MHC class I molecules were observed in states 1 to 3; ICAM1 and VCAM1 were higher in SFs of states 1 to 3, while ICAM2 was higher in state 5 (for OA, in both state 2 and state 5). Supplementary Figure 4: GSEA analysis among states 1, 3, and 5 (A) and between RA and OA SFs across states 1 to 5 (B). (A) Similar enrichment patterns in state 5 were observed in both RA and OA SFs. The VEGF signaling pathway, the Fcγ and FcεRI signaling pathway, the ERBB signaling pathway, the calcium signaling pathway, the chemokine signaling pathway, and the cytokine-cytokine receptor interaction were enriched in state 5 in both RA and OA. In OA, terms related to proinflammatory and invasive capacity were enriched in SFs in state 3, wh [file 4080735.f1.pdf]

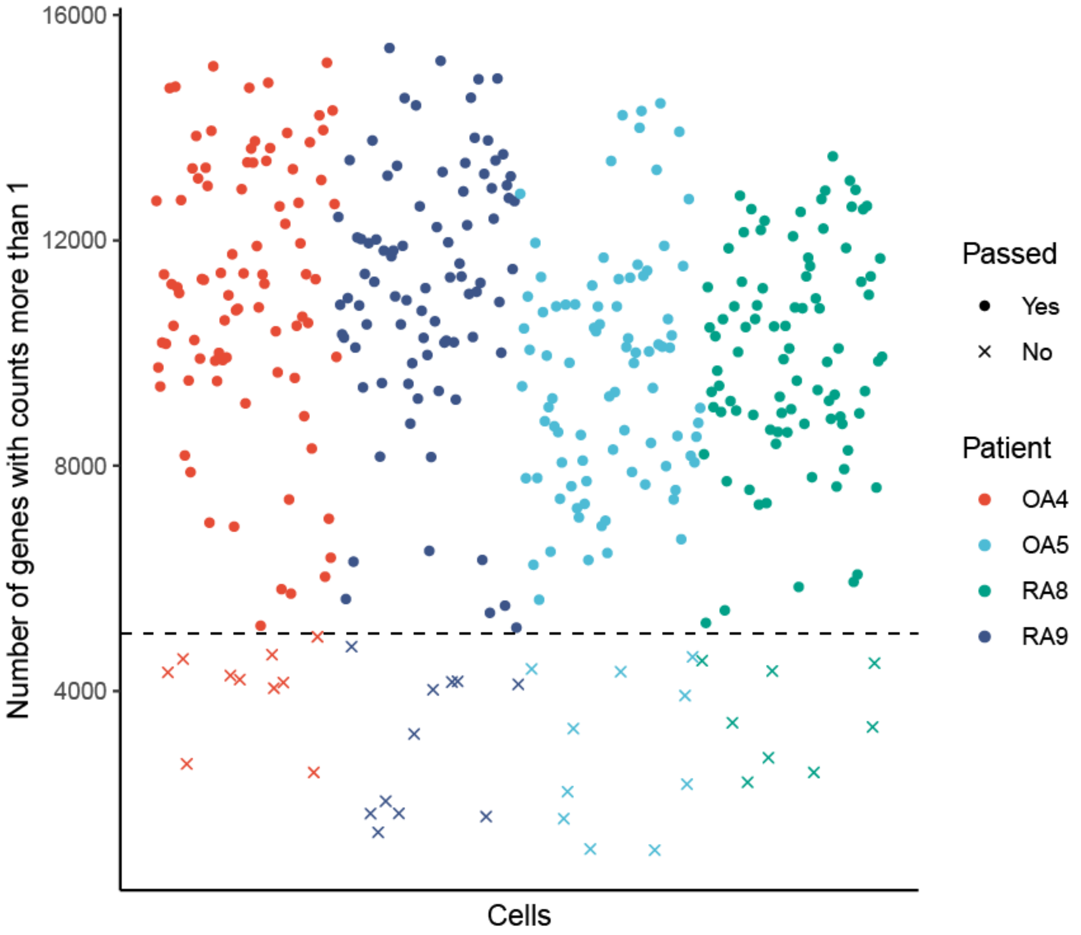

Supplementary Figure 1 Assessing quality of single-cell RNA-seq data. Numbers of expressed gene (with at least 1 read count) were calculated for all 384 SFs. 39 low-quality cells were discarded for their number of expressed genes were smaller than the threshold (dash line, medians of all cells minus 3 × median absolute deviation), and 345 cells remained. Color of plot represented its corresponding origin documented by GSE109449.

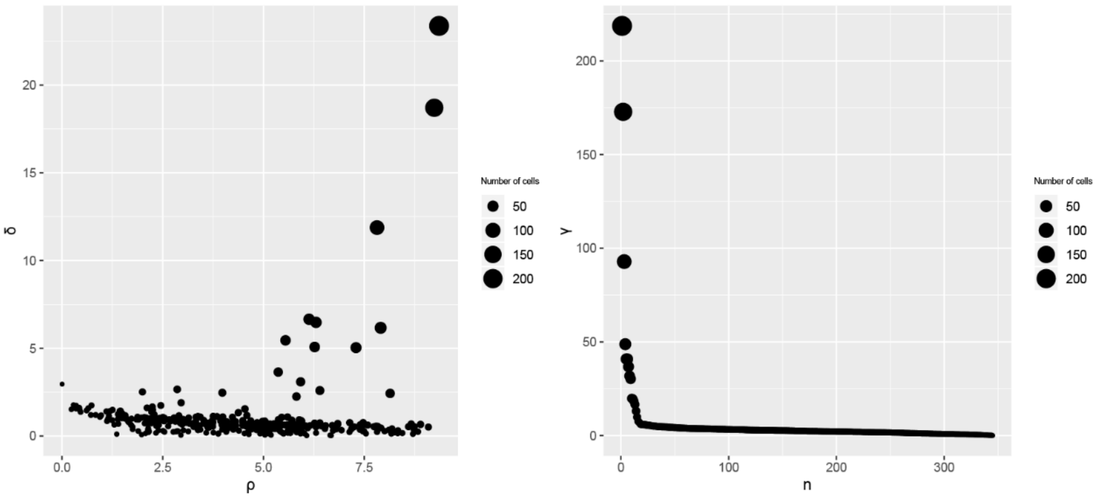

Supplementary Figure 2 Unsupervised clustering showed potential different cell clusters. According to the study from Rodriguez, A. and A. Laio (Rodriguez, A. and A. Laio, *Machine learning. Clustering by fast search and find of density peaks.* Science, 2014. **344**(6191): p. 1492-6). On the left panel, for each data point, ρ represents local density and δ represents its distance from points of higher density. On the right panel, γ value (γ=ρδ) of each points on the left panel were plotted in decreasing order.

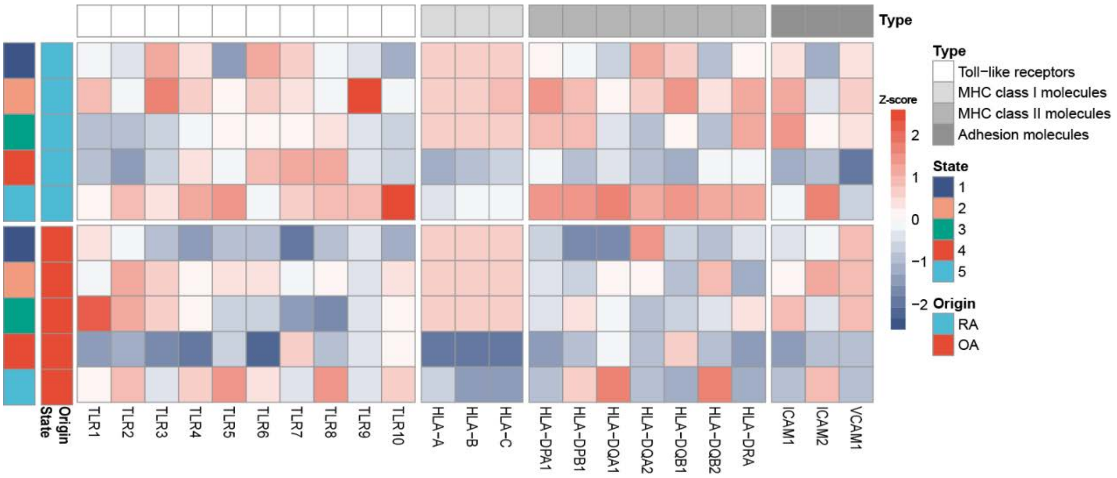

Supplementary Figure 3 Gene expression pattern of major histocompatibility complex (MHC) molecules, toll-like receptors (TLRs) and adhesion molecules across state 1 to 5 in RA and OA SFs. Higher expression level of TLRs and MHC class II molecules were observed in state 2 and 5, while of MHC class I molecules in state 1 to 3; ICAM1 and VCAM1 were higher in SFs of state 1 to 3, while ICAM2 was higher in state 5 (for OA, in both state 2 and state 5).

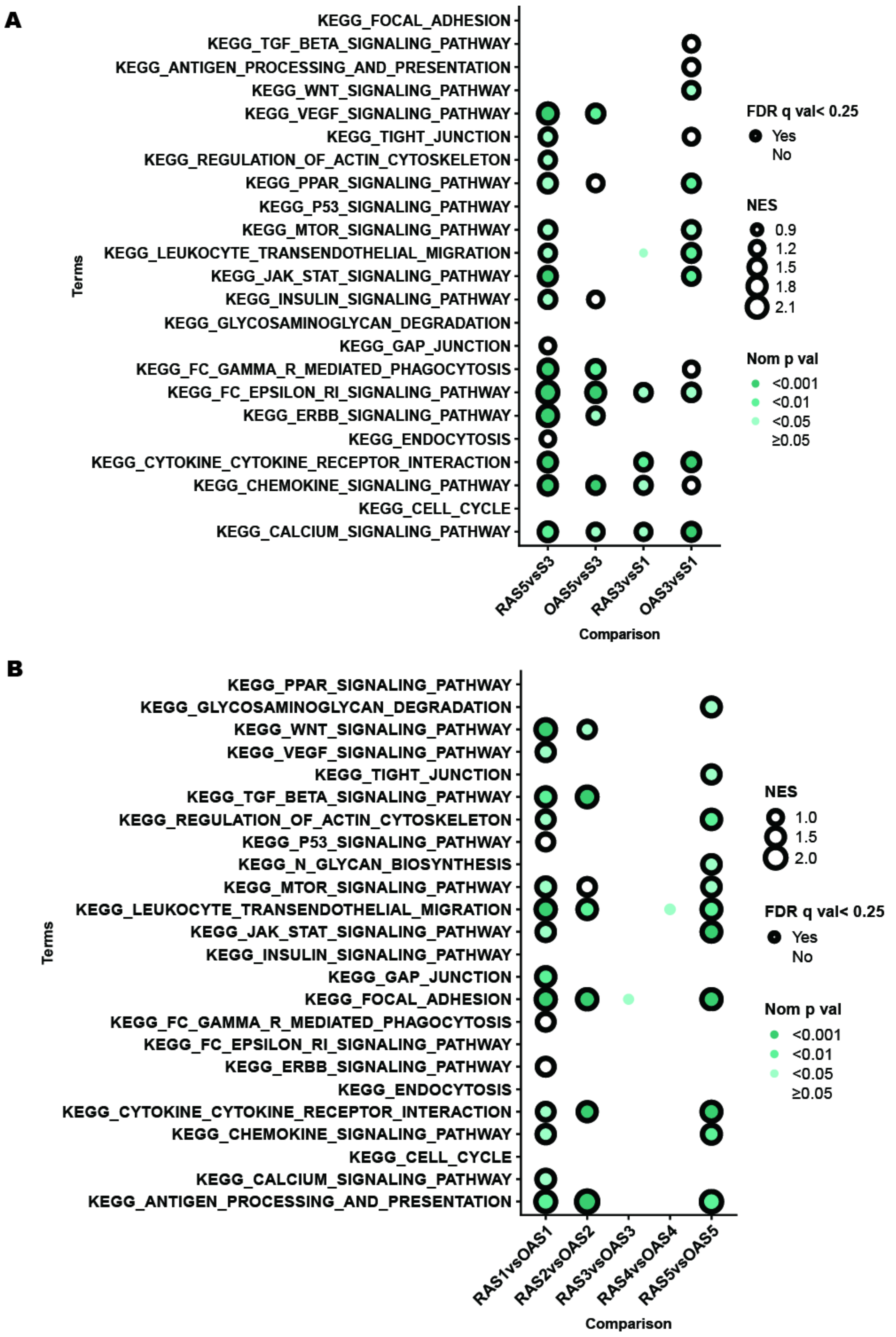

Supplementary Figure 4 GSEA analysis among state 1, 3, 5 (A) and between RA and OA SFs across state 1 to 5 (B). (A) Similar enrichment patterns in state 5 were observed in both RA and OA SFs.VEGF signaling pathway, Fcγand FcεRI signaling pathway, ERBB signaling pathway,

calcium signaling pathway, chemokine signaling pathway and cytokine-cytokine receptor interaction were enriched in state 5 in both RA and OA. In OA, terms related to proinflammatory and invasive capacity were enriched in SFs in state 3, while in RA, only genes related to FcεRI signaling pathway, calcium signaling pathway, chemokine signaling pathway and cytokine-cytokine receptor interaction were enriched in state 3. JAK-STAT signaling pathway was enriched in state 5 in RA and in state 3 in OA when compared with state 3 and state 2 respectively. (B) Except that no terms were enriched in RA SFs in state 3 and state 4, the enrichment pattern in RA SFs reflected high grade inflammation status in RA synovium.
